# An experimental test of the Community Assembly by Trait Selection (CATS) model

**DOI:** 10.1371/journal.pone.0206787

**Published:** 2018-11-30

**Authors:** Robert T. Strahan, Daniel C. Laughlin, Margaret M. Moore

**Affiliations:** 1 Biology and Environmental Science and Policy Departments, Southern Oregon University, Ashland, OR, United States of America; 2 Department of Botany, University of Wyoming, Laramie, WY, United States of America; 3 School of Forestry, Northern Arizona University, Flagstaff, AZ, United States of America; Instituto Agricultura Sostenible, SPAIN

## Abstract

The Community Assembly by Trait Selection (CATS) model of community assembly predicts species abundances along environmental gradients in relatively undisturbed vegetation. Here we ask whether this model, when calibrated with data from natural plant communities, can predict the abundances of five dominant grass species (*Bouteloua gracilis*, *Elymus elymoides*, *Festuca arizonica*, *Muhlenbergia montana*, and *Poa fendleriana*) in a greenhouse experiment that manipulated light and soil properties. To address this question, we used generalized additive models (GAMs) to model community-weighted mean (CWM) seed mass, mean Julian flowering date, and specific root length (SRL) as non-linear functions of two environmental variables (soil pH and pine basal area) in natural vegetation. The model-fitted CWM traits were then used as constraints in the CATS model to predict the relative abundance of the five grass species that were seeded in a mixture at equal densities into a 2×2 factorial experiment with soil parent material and light level as crossed factors. Light was the most important factor influencing seedling community composition, especially the abundances of *Bouteloua gracilis* and *Poa fendleriana*. The model-predicted relative abundances were significantly correlated with the observed relative abundances, and the model accurately predicted the dominant species in every treatment. *P*. *fendleriana* was correctly predicted to be the most abundant species in both shade treatments and the sun-basalt treatment, and *B*. *gracilis* was correctly predicted to be the most abundant species in the sun-limestone treatment. Our results provide experimental evidence that environmental filtering of the species pool occurs in the early stages of community assembly (including germination, emergence, and early growth), and that trait-based models calibrated with data from natural plant communities can be used to predict the outcome of the early stages of community assembly under experimental conditions.

## Introduction

Trait-based models of community assembly by environmental filtering assume that species with functional trait values that confer the highest probability of survival, growth, and reproduction in a given environment will be the dominant species in that environment [1–3]. Determining how trait values vary along environmental gradients is fundamental to developing our understanding of trait-based community assembly [4]; however, the generality of using trait-environment relationships to predict community composition has not been thoroughly assessed. In this paper, we test whether the predictions of a trait-based model of community assembly calibrated in natural vegetation can accurately predict the abundances of five dominant grasses under experimental conditions.

The theory of ‘community assembly by trait selection’ (CATS) was translated into a mathematical model by Shipley et al. (2006)[5]. The objective of the CATS model is to find the distribution of species relative abundances (*p*_*i*_) that satisfies the equality constraint: $\sum_{i=1}^{S}p_{i}t_{i}=\bar{T}$, where *t*_*i*_ represents a vector of species trait values, and $\bar{T}$ represents a community-weighted mean (CWM) trait. $\bar{T}$ represents the trait value expressed by an average individual in a community. Given that species possessing these trait values will be more abundant, a CWM trait will be biased towards trait values conferring higher fitness [5] A thorough description of the CATS model can be found in Shipley et al. 2006 [5].

Following its formulation, the CATS model has been evaluated in a variety of vegetation types [6]. Although the majority of studies have focused primarily on model performance and assessing limitations [5,7–10], application of the CATS model has been extended to forecast shifts in species distributions under climate change and to understand the relative importance of traits during community assembly [11–13]. These applications of the CATS model provide compelling evidence of trait-based environmental filtering in natural plant communities. However, most of these studies did not cross-validate the model predictions in other sites that were independent from model calibration [14]. Laughlin et al. (2011)[11] used a data-splitting approach where half of the data were used to fit trait-environment regression models, and independent predictions of community composition were made on the other half of the data; however, these predictions were not made in different spatial scales [15] or in controlled experimental conditions.

In addition to lacking independent experimental confirmation of model predictions, previous tests of the CATS model have generally focused on predicting the composition of naturally established vegetation. This approach assumes that distributions of functional traits along the environmental gradients at a single point in time provide adequate information about the dynamics of environmental filtering. However, environmental filtering of the species pool can occur during the germination-phase (i.e., radicle emergence from a seed), the emergence-phase (i.e., emergence of seedling from the soil), and the early growth phase (i.e. growth of seedling during first few weeks), when individual plants experience the conditions of the environment for the first time. We acknowledge that species differ strongly in their requirements to break dormancy to trigger germination, but our study does not address these differences directly. Species are also known to exhibit strong differences in their germination and emergence requirements [16]. Germination and emergence response are known to be influenced by both light quality (i.e., red: far-red ratio) and quantity (i.e., photosynthetic active radiation, PAR) [17]. Higher red: far-red ratios can trigger positive germination and emergence responses in light-demanding species [18], and yet greater germination and emergence rates have also been found for species exposed to lower PAR [6]. Soil water content can also influence rates of germination and emergence among species [8].

To the best of our knowledge, the predictions of the CATS model have not been tested at the early yet critical phases of germination, emergence, and early growth under differing environmental conditions. Can the CATS model, when calibrated with data from established natural vegetation; predict the relative abundance of dominant species in an experiment that manipulates light and soil properties? Here we quantify the environmental filters using trait-environment relationships observed in natural ponderosa pine forest communities of northern Arizona [10]. We then apply the CATS model in a controlled greenhouse experiment and predict the relative abundances of five grass species under controlled conditions where light and soil properties were manipulated in a crossed 2×2 factorial experiment (S1 Appendix).

## Materials and methods

### Model development: Vegetation samples, plant traits, and environmental conditions

We began by developing models to describe variation in CWM trait values as functions of environmental gradients. CWM trait values were calculated from field data, which consisted of herbaceous plant communities occupying a series of 96 permanent 1 m^2^ quadrats located across a 700 km^2^ landscape surrounding Flagstaff, Arizona, USA. These quadrats are distributed within a ponderosa pine forest ecosystem between the elevations of 2000–2500 m and span a range of soil parent material developed from basalt and limestone parent material. Dense stands of ponderosa pine are interspersed with fragmented patches of grass-dominated openings. The understory herbaceous plant community, dominated by C_3_ and C_4_ perennial bunchgrasses followed by perennial, annual and biennial forbs, is an integral component of this ponderosa pine forest and is the major source of plant diversity [19].

We used three functional traits measured on all 79 herbaceous species found on the quadrats, including seed mass, specific root length (SRL) and mean Julian flowering date (flowering date) (S3 Appendix)[20]. Average trait values were obtained for each species using standardized methods as described in S4 Appendix. To determine the relationship between functional traits and environmental conditions in natural vegetation, we calculated community-weighted mean (CWM) trait values (i.e., $\bar{T}=\sum_{i=1}^{S}p_{i}t_{i}$, where *p*_*i*_ are relative abundances and *t*_*i*_ are trait values of species *i*) for each functional trait. Observed relative abundances (i.e. *p*_*i*_) were calculated using visual estimates of foliar cover for all species in each of the ninety-six 1 m^2^ quadrats using standard vegetation sampling techniques [21]. Quadrats were visually divided into 1% squares and professional botanists estimated the total number of 1% squares occupied by each species. Relative abundance of each species in a quadrat were calculated by dividing the percent of each species by the total percent cover of the quadrat. Visual estimates were made during a two-month period between August and September of 2011. We chose to analyze three traits measured on three different plant organs (seeds, flowers, and roots) because including multiple traits from multiple organs has been shown to improve trait-based predictions of community composition [7].

Seed mass is the oven-dry mass of an average seed of a species expressed in mg [22]. Seed mass reflects a fundamental tradeoff between seed size and reproductive output. Larger seeded species produce fewer seeds for a given reproductive effort, yet seedlings have more reserves to establish in low-resource environments [23]. This is an important characteristic as well because in dense shade larger seeds are expected to contribute to longer survival during the cotyledon stage owing to greater metabolic reserves [24].

SRL is the ratio of fine root (< 2mm diameter) length to dry mass expressed as m g^-1^ and reflects foraging potential relative to carbon investment [22]. SRL has been shown to be positively correlated with relative growth rate [25] and with higher rates of nutrient acquisition [26].

Flowering date reflects the mean Julian date that a species flowers and was determined for each species using regional floras [20]. Flowering date represents a species’ life history strategy related to phenological timing of germination and emergence, reproduction, and growth [27,28]. Germination and emergence responses are largely regulated by expression of the flowering time gene Flowering Locus C (FLC) [29,30]. C_3_ grasses generally have earlier flowering dates than C_4_ grasses in this ponderosa pine bunchgrass ecosystem. Using flowering date as opposed to the C_4_ and C_3_ trait explicitly, better represents the variability that exists between these species in terms of phenology.

We used values of soil pH and pine basal area measured at each of the 96 quadrats. Pine basal area represents the cross-sectional area of ponderosa pine trees within a given area, expressed as m^2^ ha^-1^. It was measured in a 20 × 20 m plot centered on each quadrat as described in Laughlin 2011 [30]. Soil pH was measured at each quadrat between July and August as described in Laughlin et al. (2011) [31]. Soil pH and pine basal area are two environmental variables that have been shown to be good predictors of plant community composition [32] and trait distributions [10,30]) in this ponderosa pine-bunchgrass vegetation. Soil pH has important implications for community composition through its influence on nutrient availability and species-specific tolerances to acidic or basic conditions [33]. Pine basal area impacts community composition directly by altering light availability to the forest floor [34], through belowground competition for soil moisture [35], and indirectly through litter fall of recalcitrant needles, decreasing soil pH and altering the availability of nutrients in the mineral soil [31].

Generalized additive models (GAMs) were developed using soil pH and pine basal area to model the variation in each of the three CWM traits (seed mass, SRL, and flowering date) across the 96 quadrats. GAMs were fit with a Gaussian error distribution and a maximum smoothing value of 10 using the ‘gam’ function in the ‘mgcv’ library of R [36].

### Greenhouse experiment

The experiment was conducted over a three-month period (August–November 2011) in the Greenhouse Research Complex at Northern Arizona University in Flagstaff, Arizona, USA. We focus on the five most abundant grass species found across our quadrats, including two C_4_ grass species (*Bouteloua gracilis* (Wild. Ex Kunth) Lag. Ex Griffiths, and *Muhlenbergia montana* (Nutt.) Hitchc.) and three C_3_ grass species (*Elymus elymoides* (Raf.) Swezey, *Festuca arizonica* (Vasey), and *Poa fendleriana* (Steud) Vasey). These species represent the broad spectrum of functional trait variation that exists among native grass species in this ecosystem (S2 Appendix). We chose to test the model using the these five species for two reasons: 1) These grasses dominate the understory, contributing approximately 70% of the total biomass of herbaceous plants in this ecosystem (21). These species are of particular interest to managers because they represent an important forage base for livestock and wildlife and are integral to the high frequency, low intensity surface fires characteristic of these ponderosa pine forests [37,38].

We established a 2 × 2 factorial study with soil parent material and light level as crossed factors. Soils derived from limestone and basalt substrates were chosen because they reflect significant differences in soil pH across our study site. Limestone and basalt-derived soils were collected from two sites on the Coconino National Forest near Flagstaff, Arizona. All soils were double sterilized prior to use in a 0.19 cubic meter electric sterilizer. Forty 3.78-liter pots were filled with sterilized limestone-derived soil and 40 one-gallon pots were filled with sterilized basalt-derived soil.

Pine basal area is an important driver of light gradients in our system. Two contrasting light levels were chosen to reflect the lowest and highest pine basal area found across our study; light quantity in full sun where pine basal area is zero m^2^ ha^-1^ and dense shade where pine basal area is 59 m^2^ ha^-1^. While light levels were used as surrogates for pine basal area in our experiment, values of pine basal area were used to make CATS model predictions (described below). To replicate light conditions in the greenhouse we made comparisons of light conditions between the field and greenhouse, a Sunfleck Ceptometer (Decagon, Pullman, WA), was used to measure photosynthetic photon flux density (PPFD: 400–700 nm), which indicates the amount of photosynthetic active radiation (PAR) falling on a given surface. These measurements were made on cloud free days, between 11:00 and 14:00 Mountain standard time at approximately 30 cm above the ground during July-August of 2011. Average PPFD in full sun corresponded to an average of 2149 μmol m^-2^ s^-1^, while deep shade average 74.8 μmol m^-2^ s^-1^. For our shade treatment we used stock shade cloth designed to block out 50% of available light. This resulted in an average daily PPFD on the shade treatments of 83.4 μmol m^-2^ s^-1^, approximating the value of 74.8 μmol m^-2^ s^-1^ under deep shade. Full sun treatments had an average greenhouse PPFD of 909 μmol m^-2^ s^-1^. This reduction can be attributed to the greenhouse glass and corresponds to approximately 30 m^2^ ha^-1^ pine basal area.

Shade treatments were applied to half of the pots for each parent material; the shade cloth was hung approximately one meter above the pots. The remaining pots were placed in full light. Twelve seeds of each species were raked into the soil of each pot; this resulted in an average sowing density of 0.13 seeds cm^-2^ (1,300 seeds m^-2^), which is comparable to average seeding densities found in open canopy forests in this ecosystem [39]. Given the equal sowing densities for each species, no species was seed or dispersal limited in this experiment. Pots were randomly arranged on the greenhouse benches and were watered equally once daily. No pots received fertilizer treatments, and weeds were removed daily. Throughout the duration of the study no germination-emergence was observed in three communities in each light treatment containing limestone soil and four communities in each light treatment containing basalt soil, so we removed these pots from the analysis. This left a total of 66 pots in the analysis; 17 in both sun and shaded limestone treatments and 16 in each sun and shaded basalt treatments. We tracked the germination-emergence and survival of all individuals of each species in every pot once every two weeks for the duration of the project, which lasted 80 days. Final counts were used to calculate species relative abundances by density, which were then used to test the model.

### Model predictions

GAMs developed from field data (described in *Model development* section above) were used to make predictions of optimal CWM trait values under greenhouse conditions. To make predictions we used values of soil pH and pine basal area collected from across our 96 quadrats [30,31]. For limestone soils we used mean values of 6.8 for soil pH and for basalt soils we used mean values of 5.9 for soil pH. For pine basal area we used zero m^2^ ha^-1^ to represent our ‘sun’ treatment and 59 m^2^ ha^-1^ to represent our ‘shade’ treatment. We chose to use zero m^2^ ha^-1^ instead of the 30 m^2^ ha^-1^ because this approach is most consistent with how we designed the study and zero m^2^ ha^-1^ yielded better predictions.

Predictions of CWM trait values were then used as constraints in the CATS model to predict the relative abundance of each of the five grass species in the experimental communities. Predictions for CWM traits in each treatment are summarized in S5 Appendix.

The predictions of relative abundances used all three CWM traits simultaneously. Given that there were fewer constraints than species, this system of linear equations will have many possible solutions. The CATS model chooses the probability distribution (i.e., distribution of relative abundances, *p_i_*) that maximizes the entropy function, $-\sum_{i=1}^{S}p_{i}lnp_{i}$ (i.e., the distribution is maximally even subject to the linear constraints). The constraints are the CWM traits ($\bar{T}$) are constants that constrain the solutions of species abundances. Therefore, we obtained predictions of relative abundance for each of the five species in each of the four (2 × 2) treatment combinations.

### Statistical analysis

We examined the main effects of light and soil parent material, and the light × soil interaction effect on resulting community composition and observed CWM traits. We used a two-way factorial permutation-based multivariate analysis of variance (PerMANOVA, [40]where Bray-Curtis dissimilarity was used as the distance measure for multivariate data (community composition) [41]. The Euclidean distance measure was used for univariate data (individual species). All analyses were performed using the ‘vegan’ library [42] of R version 3.2.2 [43].

To evaluate the predictive performance of the CATS model under experimental conditions, we examined the degree of correlation between the observed and predicted vegetation structure and CWM traits. We compared the average observed relative abundances for each species and CWM trait in each treatment to the predicted relative abundances and CWM trait for each treatment combination. This resulted in a 4 × 5 “treatment × species” and 4 × 3 “treatment × CWM trait” matrix.

Model predictions were compared with observed relative abundances using two measures of fit: *R*^2^ (using untransformed relative abundances) and the Root Mean Square Error (RMSE_sqrt_) using square-root transformed relative abundances [44]. We evaluated the statistical significance of model predictions by comparing measures of fit obtained from predicted constraints against a null distribution of 999 measures of fit obtained by permuting observed relative abundances. Concordance between the predicted and observed relative CWM trait values was evaluated by correlation analysis with Pearson’s correlation coefficient.

## Results

### Model development: Vegetation samples and environmental conditions

The environmental variables explained between 15–44% of the variance of the three CWM traits (Table 1). Soil pH and pine basal area were both significant factors for explaining variation in seed mass, SRL, and flowering date.

**Table 1:** Predictive models of community-weighted mean traits as a smoothed function of soil pH and pine basal area (Pine BA).

Community-weighted mean trait	pH	Pine BA (m^2^ ha^-1^)	Model *R*^2^ (adjusted)
Seed mass	* ^(-)^	** ^(+)^	0.15
Specific root length	*** ^(+)^	*** ^(-)^	0.44
Flowering date	** ^(+)^	*** ^(-)^	0.38

Cubic-spline regression smoothers (Generalized Additive Models) were used to fit the models using the “mgcv” package in R.

Asterisks indicate significance level and signs in parentheses indicate direction of change.

Signif. codes: 0.0001 ‘***’, 0.001 ‘**’, 0.01 ‘*’, 0.05 ‘.’, 0.1 ‘blank’

### Treatment effects

**Fig 1:**
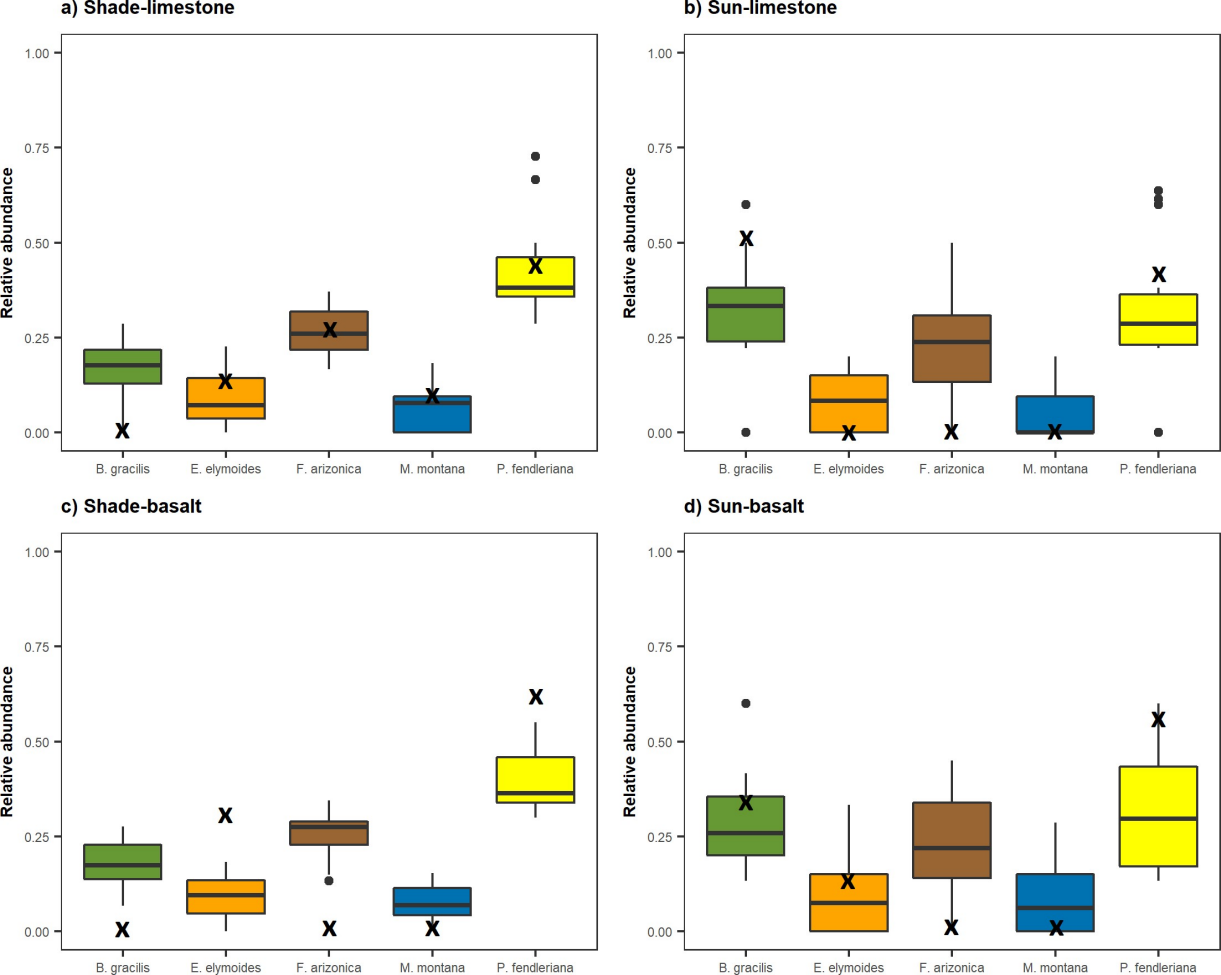
Distribution of observed (boxplots) and predicted (**x**) relative abundances of *B*. *gracilis* (green), *E*. *elymoides* (orange), *F*. *arizonica* (brown), *M*. *montana* (blue), and *P*. *fendleriana* (yellow) in four treatments based on parent material and light level; a) shade-limestone, b) sun-limestone, c) shade-basalt, and d) sun-basalt.

Survival was close to 100% for most species in each treatment, however germination and emergence varied greatly among species and treatments (data summarized in S6 Appendix). Light explained a significant amount of variation in overall seedling community composition (*R*^2^ = 0.14) (Table 2). There was no significant main effect of soil parent material or light × soil parent material interaction on community composition (Table 2). Light was also the only factor that explained a significant amount of variation among species (Table 2). For *B*. *gracilis*, light explained 30% of the variability in its relative abundance. *B*. *gracilis* was slightly more abundant on limestone compared to basalt soils and it performed best in high light (Fig 1). Light was a significant factor influencing the distribution and abundance of *P*. *fendleriana* and explained 12% of the variability in its observed relative abundance. *P*. *fendleriana* was observed to be the dominant species in shade treatments and performed slightly better on limestone parent material (Fig 1B). Seedling abundances of *E*. *elymoides*, *F*. *arizonica*, or *M*. *montana* were not influenced by any treatment (Table 2).

**Table 2:** Results from a two-way PerMANOVA with light (sun, shade), parent material (basalt, limestone), and their interaction as factors influencing the resulting species abundances in the experiment.

Source	*F*_1,62_	*R*^2^	*P*
Community			
Light	10.2	0.14	< 0.001*
Soil parent material	0.47	0.00	0.70
Light × soil interaction	0.22	0.00	0.85
*B*. *gracilis*			
Light	26.3	0.30	< 0.001*
Soil parent material	0.64	0.01	0.43
Light × soil interaction	0.90	0.01	0.36
*E*. *elymoides*			
Light	0.04	0.00	0.86
Soil parent material	0.13	0.00	0.71
Light × soil interaction	0.00	0.00	0.95
*F*. *arizonica*			
Light	2.04	0.03	0.15
Soil parent material	0.00	0.00	0.97
Light × soil interaction	0.18	0.00	0.67
*M*. *montana*			
Light	0.00	0.00	0.97
Soil parent material	1.85	0.03	0.18
Light × soil interaction	0.33	0.00	0.57
*P*. *fendleriana*			
Light	8.82	0.12	0.003*
Soil parent material	0.07	0.00	0.79
Light × soil interaction	0.02	0.00	0.88

‘Community’ refers to the relative abundances of all five species as a multivariate response.

Asterisks (*) indicate significance level.

Light was the only factor explaining a significant amount of variability in observed CWM trait values in the experimental communities (Table 3). Light explained 22% of the variation in CWM SRL and 23% of the variation in CWM flowering date. No significant effect of light, parent material, or their interaction was found for CWM seed mass.

**Table 3:** Results from a two-way PerMANOVA with light (sun, shade), parent material (basalt, limestone), and their interaction as factors influencing resulting community-weighted mean trait values in the experiment.

Source	*F*_1,62_	*R*^2^	*P*
*Seed mass*			
Light	0.00	0.00	0.97
Soil parent material	0.10	0.00	0.76
Light × soil interaction	0.00	0.00	0.99
*Specific root length (SRL)*			
Light	18.1	0.22	< 0.001*
Soil parent material	0.01	0.00	0.90
Light × soil interaction	0.28	0.00	0.60
*Flowering date*			
Light	18.7	0.23	< 0.001*
Soil parent material	0.01	0.00	0.92
Light × soil interaction	0.31	0.00	0.59

Asterisks (*) indicate significance level.

### Model predictions

**Fig 2:**
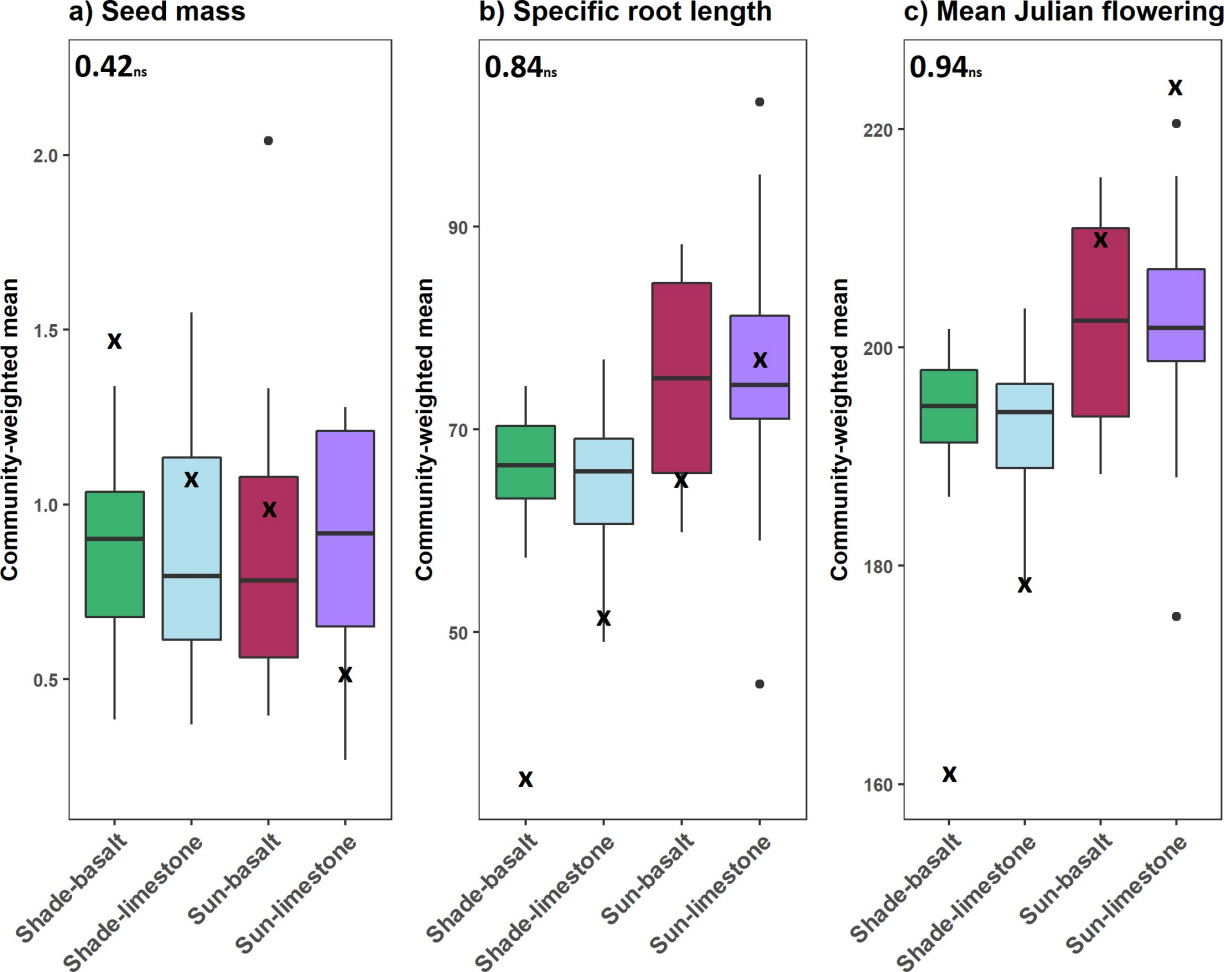
Distribution of observed (boxplots) and predicted (**x**) CWM trait values for a) Seed mass, b) Specific root length, and c) Mean Julian flowering date in four treatments based on light level and parent material; shade-basalt (green), shade-limestone (red), sun-basalt (blue), and sun-limestone (purple). Pearson’s correlation coefficients are given. No significant (ns) correlations between observed and predicted CWM trait values were found.

Fig 2 illustrates the relationship between model-predicted relative abundances (the ×’s) versus the range of observed relative abundances among the replicate experimental communities (box plots) (data can be found in S7 and S8 appendices). The predicted relative abundances of species were significantly correlated with the mean observed relative abundances across treatments (*R*^*2*^ = 0.53, *P* = 0.0010; *RMSE*_sqrt_ = 0.25, *P* = 0.0045). The CATS model correctly predicted the most abundant species in each treatment (although the observed abundance of *B*. *gracilis* was only slightly more abundant than *P*. *fendleriana* in sun-limestone treatments; 33% vs 31% respectively), and in 72% of the experimental greenhouse communities. *P*. *fendleriana* was correctly predicted to be the most abundant species in both shade treatments and the sun-basalt treatment (Fig 1). *B*. *gracilis* was correctly predicted to be the most abundant species in sun-limestone treatments. *F*. *arizonica* and *B*. *gracilis* were correctly predicted to be the second most abundant species in shade-limestone and sun-basalt treatments, respectively (Fig 1A and 1D). The model also correctly predicted *P*. *fendleriana* to be the second most abundant species following *B*. *gracilis* in sun-limestone treatments. *F*. *arizonica* was consistently more abundant than *E*. *elymoides*, however the model was only able to predict this ranking in the shade-limestone treatments.

Fig 2 illustrates the relationships between the GAM predicted CWM traits and the range of observed CWM trait values among sample units. We found no significant correlations between predicted CWM trait values and the observed mean CWM trait values in each treatment (Fig 2). For each trait, the predicted values showed trends across light and soil conditions, while no such trend is visible in the observed CWM traits. However, the model predictions for SRL and flowering date were reasonable, for example higher CWM SRL and later flowering dates were predicted in sun versus shade treatments (Fig 2B and 2C). Model predictions were poorest for CWM seed mass (data can be found in S4 Appendix).

## Discussion

This study examined the generality of a trait-based model by predicting the outcome of the early phase of community assembly under experimental conditions. Using seed, root, and flowering traits, the CATS model, calibrated in natural communities predicted different abundances of common grass species among the different treatment combinations of light and soil parent material. Our results have two main implications for vegetation science. First, environmental filtering is an important process in the early stages of community assembly, but light exhibited stronger effects on the germination, emergence, and early growth phases than soil parent material. Second, the trait-based CATS model can produce predictions that are significantly correlated with observations under experimental conditions that are independent from the locations in which the model was calibrated, but accuracy varied among species and treatment combinations.

### Environmental filters on germination, emergence, and early growth

On small spatial scales, where species are not limited by dispersal, the community assembly process reflects the influence of environmental filters and biotic interactions [45]. While many important processes control the assembly of species (i.e., dispersal limitation, demographic stochasticity, positive and negative species interactions, disturbance), this study isolated the early stages of germination, emergence, and early growth while eliminating the possibility of dispersal limitation. Nearly 100% of the individual seedlings that germinated survived until the end of the experiment. Yet species differed strongly in their germination and emergence rates, indicating that the environmental conditions affecting seed germination and emergence were important filters influencing the assembly of the experimental communities.

A substantial amount of research documents the importance of light in the germination and emergence phases of plant development. The primary mechanism relates to how plants respond to changes in the spectral ratio of red and far-red light [46,47]. This mechanism has been shown to be more important for smaller seeded species suggesting an increased sensitivity to microsite availability related to shading and competition [18,47,48]. This likely relates to an increased risk of mortality for smaller seeded species compared to larger seeded species when subject to post germination hazards and the need to optimize resource acquisition [49]. Light was not a significant factor for the larger seeded species in our study, *E*. *elymoides* and *F*. *arizonica*, suggesting a capacity to utilize seed reserves that allowed them to occupy a wider range of microsites as well as buffer post-germination hazards. Previous research also documents that this response is species-specific, which agrees with our findings [50]. For example, while both *P*. *fendleriana*, and *B*. *gracilis* have small seeds and showed a significant response to the light treatment in our experiment, *M*. *montana*, the species with the smallest seed mass did not. Furthermore, the experimental treatments had no significant effect on observed CWM seed mass, indicating that seed mass does not have clear predictable effects on germination, emergence, and early growth across the range of environmental conditions studied here.

Full sun treatments reflect high resource environments, which have been shown to select for species that tend to have high SRL and later flowering dates [25]. We expected the two C_4_ species (i.e. *B*. *gracilis* and *M*. *montana*), which have high SRL and a late flowering to perform best in sun treatments because at higher temperatures and greater light intensities SRL would allow for a more rapid acquisition of nutrients and a later flowering date is associated with species better adapted to persist in warmer, high light conditions [30,33]. Yet, only *B*. *gracilis* performed well in our full sun treatments. In addition, P. *fendleriana*, a C_3_ species with low SRL, and an early flowering date performed nearly as well as *B*. *gracilis* in these treatments, suggesting that in high resource environments these traits do not necessarily confer a competitive advantage during early stages of community assembly. In contrast, shade treatments reflect resource-poor environments, so species with traits that confer an ability to tolerate low light levels would be expected to be dominant [51,52]. Our findings were more consistent with this. For example, all three C_3_ species (*E*. *elymoides*, *F*. *arizonica*, and *P*. *fendleriana*), with relatively low SRL and early flowering dates performed best in shade treatments. Decreases in light availability associated with increased ponderosa pine density has been correlated with a shift in understory community composition towards species exhibiting whole-plant shade tolerant strategies associated with low SRL and earlier flowering times [32].

Flowering date relates to the phenology of life history events and exhibits pleiotropy with germination and emergence in some species [36,53]. Specifically, the major flowering time gene Flowering Locus C (FLC) has been shown to regulate temperature dependent germination and emergence [36]. Flowering date differs most between the C_3_ and C_4_ species in our study and all three C_3_ species tended to be more abundant in shaded treatments. While we do not not know the degree of FLC expression among these species it suggests a possible mechanism for this finding. Earlier germination and emergence can also provide a competitive advantage, enabling plants to reach a larger size before reproduction, thereby increasing fecundity [54]).

### Model predictions

Predicted and observed CWM trait values were not significantly correlated. We did observe general agreement in at least one treatment for each trait. For example, there was general agreement between predicted and observed CWM seed mass in shade-limestone and sun-basalt, but not shade-basalt or sun-limestone. There was also general agreement between observed and predicted CWM SRL in sun-basal and sun-limestone and for flowering date in sun-basalt treatments. Higher CWM SRL and flowering dates in sun versus shade is also consistent with the trait-environment relationships shown in the fitted GAMs, which reflect the observed trait distributions in the natural plant communities. This is important because the CATS model, despite the imperfect prediction of CWM traits in the experiment, still yielded reasonable predictions of species relative abundances. In this case, the agreement found between observed and predicted CWM trait values in at least one treatment for each trait compensated for poorer CWM trait predictions elsewhere and their influence on CATS model predictions of relative abundances.

Predictive accuracy can also be attributed to several factors related to CATS model constraints and the experimental environment. Similarity in trait values among species in a given community has been shown to decrease the predictive accuracy of the CATS model [8]. Greater similarity in trait values can increase the number of feasible solutions to the system of linear equations, so there would be a larger set of distributions that agree with the constraints. While these species represent a broad range of trait values among grass species in this ecosystem, their range of traits with respect to other understory species (e.g., legumes, annual forbs, etc.) is restricted. Also, our measure of relative abundances based on density might be sufficiently distinct from the visual estimates of cover that were used to calibrate the trait-environment relationships to impact CATS model predictions. It cannot be overlooked that the CWM traits used as constraints to predict the relative abundance of our five grass species were predicted from field data. So, while we assume that similar traits will manifest in similar environments, differences in soil pH and light availability across each of our experimental communities likely represented sufficiently different environments from those natural communities in which CWM traits were estimated. Measuring soil pH in each experimental community might have allowed for more precise predictions, rather than using mean values that were calculated from field data throughout the region.

The validity of any scientific theory rests in its ability to make accurate and generalizable predictions [55]. By providing quantitative predictions of the relative abundances of individual species in a community, the CATS model can be used to directly test the theoretical framework of trait-based community assembly through environmental filtering. Our results provide evidence that environmental filtering and species sorting occurs in the phases of germination, emergence, and early growth, and that trait-based models can predict the outcome of these early stages of community assembly under experimental conditions. Additional experimental studies will be useful for examining the predictive ability of trait-based models using more functionally diverse communities at various spatial and temporal scales.

## Supporting information

S1 Appendix Summary of our general approach. (DOCX)

S2 Appendix Bivariate scatterplots illustrating functional trait variation among the 16 most common graminoid species across the study site. (DOCX)

S3 Appendix Average values for three functional traits for each of the 79 herbaceous species measured on 96 permanent 1-m2 quadrats. (DOCX)

S4 Appendix Trait measurement methodology. (DOCX)

S5 Appendix Predictions for CWM traits in each treatment. (DOCX)

S6 Appendix Survival and germination-emergence rates for species in each treatment. (DOCX)

S7 Appendix Observed relative abundance of each species in each treatment. (XLSX)

S8 Appendix Predicted relative abundance of each species in each treatment. (XLSX)

## Abbreviations

CWM: Community-weighted mean trait value
CATS: Community assembly by trait selection
SRL: Specific root length
