# Supplementary material for: An experimental test of the Community Assembly by Trait Selection (CATS) model
Source: PLoS One. 2018 Nov 30;13(11):e0206787. doi: 10.1371/journal.pone.0206787 (PMC6267976; doi:10.1371/journal.pone.0206787)
Supplement: S1 Appendix — (DOCX) [file pone.0206787.s001.docx]

S1 Appendix: Supporting information to the paper

Strahan, R.T. et al. An experimental test of the Community Assembly by Trait Selection (CATS) model


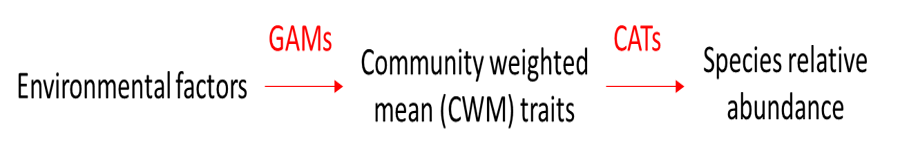


**S1 Appendix**. Summary of our general approach. We used generalized additive models (GAMs) to model community-weighted mean (CWM) traits as non-linear functions of two environmental factors in natural vegetation. The model-fitted CWM traits were then used as constraints in the CATS model to predict the relative abundance of five grass species.
