# Supplementary material for: An experimental test of the Community Assembly by Trait Selection (CATS) model
Source: PLoS One. 2018 Nov 30;13(11):e0206787. doi: 10.1371/journal.pone.0206787 (PMC6267976; doi:10.1371/journal.pone.0206787)
Supplement: S2 Appendix — (DOCX) [file pone.0206787.s002.docx]

S1 Appendix: Supporting information to the paper

Strahan, R.T. et al. An experimental test of the Community Assembly by Trait Selection (CATS) model


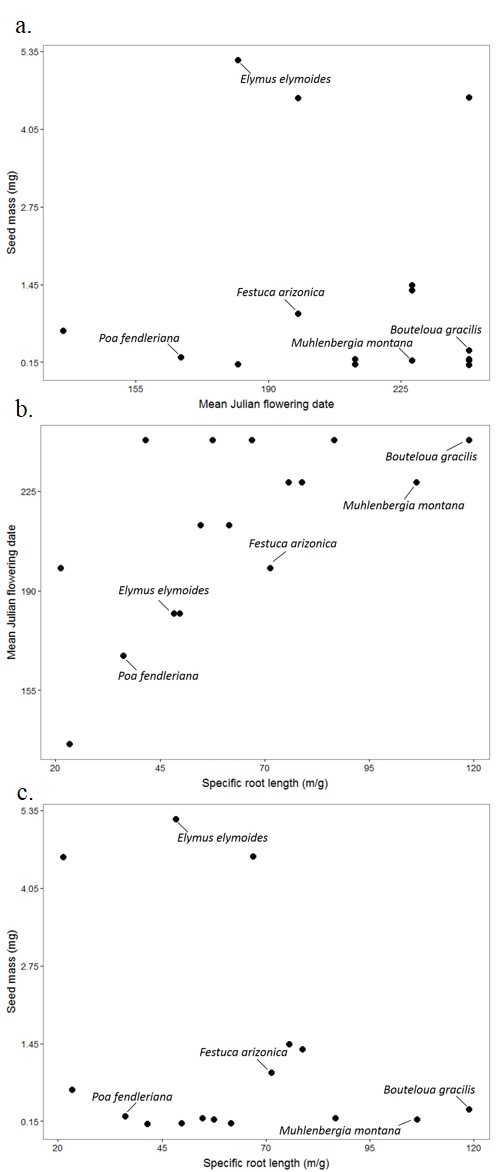


**S1 Appendix**. Bivariate scatterplots of the 16 most common graminoid species

across the study site; a) mean Julian flowering date and seed mass, b) specific root length and

mean Julian flowering data, and c) specific root length and seed mass data
