# Supplementary material for: An experimental test of the Community Assembly by Trait Selection (CATS) model
Source: PLoS One. 2018 Nov 30;13(11):e0206787. doi: 10.1371/journal.pone.0206787 (PMC6267976; doi:10.1371/journal.pone.0206787)
Supplement: S3 Appendix — (DOCX) [file pone.0206787.s003.docx]

S2 Appendix: Supporting information to the paper

Strahan, R.T. et al. An experimental test of the Community Assembly by Trait Selection (CATS) model

**S2 Appendix**. Average values for three functional traits for each of the 79 herbaceous species measured on 96 permanent 1 m^2^ quadrats. These trait values were used to calculate community-weighted mean traits based on foliar cover estimates of the 79 species.

| Species | Seed mass (mg) | Mean Julian flowering date | Specific root length (m g^-1^) |
| --- | --- | --- | --- |
| *Achillea millefolium* | 0.14 | 197 | 35.3 |
| *Ambrosia psilostachyia* | 5.896 | 243 | 72.9 |
| *Antennaria parvifolia* | 0.061 | 182 | 27.4 |
| *Antennaria rosulata* | 0.073 | 182 | 29.5 |
| *Arenaria fendleri* | 0.184 | 182 | 50.1 |
| *Arenaria lanuginosa* | 0.178 | 197 | 28.7 |
| *Artemisia campestris* | 0.085 | 228 | 52.8 |
| *Artemisia caruthii* | 0.054 | 259 | 66.2 |
| *Astragalus humustratus* | 1.95 | 197 | 35.7 |
| *Bahia dissecta* | 0.383 | 259 | 99 |
| *Blepharoneuron tricholepis* | 0.174 | 243 | 57.6 |
| *Boutelous gracilis* | 0.34 | 243 | 118.9 |
| *Bromus ciliatus* | 4.575 | 243 | 67 |
| *Carex geophila* | 0.673 | 136 | 23.4 |
| *Castilleja spp.* | 0.132 | 182 | 18.4 |
| *Chaetopappus ericoides* | 0.209 | 213 | 43.3 |
| *Chamaecyce fendleri* | 0.118 | 197 | 84.5 |
| *Chamaecyce serpylifolia* | 0.215 | 213 | 149 |
| *Chenopodium gracilis* | 0.226 | 243 | 112.8 |
| *Cirsium wheeleri* | 12.802 | 228 | 17.6 |
| *Coreopsis tinctoria* | 0.254 | 213 | 28.6 |
| *Drymaria leptophylla* | 0.033 | 243 | 153.4 |
| *Echandia flavescens* | 1.113 | 213 | 104.1 |
| *Elymus elymoides* | 5.203 | 182 | 48.4 |
| *Eriogonum alatum* | 7.079 | 213 | 27.4 |
| *Erigeron divergens* | 0.046 | 182 | 28.5 |
| *Erigeron flagellaris* | 0.038 | 182 | 42.1 |
| *Erigeron formossisimus* | 0.196 | 228 | 47.9 |
| *Eriogonum racemosum* | 2.34 | 228 | 41 |
| *Festuca arizonica* | 0.961 | 198 | 71.4 |
| Species | Seed mass | Flowering date | Specific root length |
| *Gaillardia pinnatum* | 1.594 | 167 | 59.8 |
| *Geranium caespitosum* | 1.692 | 213 | 10.7 |
| *Heliomeris multiflora* | 0.436 | 213 | 108.6 |
| *Helianthella quinquinervis* | 7.108 | 228 | 11.6 |
| *Hieracium fendleri* | 0.488 | 182 | 38.4 |
| *Houstonia wrightii* | 0.439 | 182 | 27.3 |
| *Hymenoxys richardsonii* | 0.546 | 197 | 37.2 |
| *Koeleria macrantha* | 0.11 | 213 | 61.6 |
| *Linum australe* | 0.565 | 198 | 58 |
| *Linaria dalmatica* | 0.14 | 213 | 26.6 |
| *Lotus wrightii* | 3.091 | 197 | 26.4 |
| *Lupinus argenteus* | 20.496 | 198 | 12.8 |
| *Mirabilis decipiens* | 4.528 | 228 | 45 |
| *Muhlenbergia minutissima* | 0.096 | 243 | 127.8 |
| *Muhlenbergia montana* | 0.171 | 228 | 106.4 |
| *Muhlenbergia rigens* | 0.1 | 243 | 41.5 |
| *Muhlenbergia wrightii* | 0.2 | 243 | 86.7 |
| *Nama dichotomum* | 0.059 | 243 | 183.7 |
| *Oxalis spp.* | 0.25 | 228 | 117.8 |
| *Oxytropis lambertii* | 3.536 | 182 | 10.4 |
| *Packera multilobata* | 0.419 | 198 | 21 |
| *Pascopyrum smithii* | 4.57 | 198 | 21.3 |
| *Penstemon barbatus* | 0.243 | 182 | 25.7 |
| *Penstemon linearifolium* | 1.307 | 182 | 50.3 |
| *Penstemon virgatus* | 0.893 | 213 | 36.3 |
| *Phlox speciosa* | 2.13 | 136 | 35.1 |
| *Plantago argyrrea* | 1.458 | 213 | 104.9 |
| *Poa compressa* | 0.199 | 213 | 54.8 |
| *Poa fendleriana* | 0.227 | 167 | 36.3 |
| *Poa pratensis* | 0.109 | 182 | 49.8 |
| *Polygonum douglasii* | 0.958 | 213 | 85.3 |
| *Portulaca oleracea* | 0.122 | 228 | 204.1 |
| *Potentilla crinita* | 0.949 | 182 | 44.7 |
| *Potentilla hippiana* | 0.52 | 213 | 17.8 |
| *Potentilla subviscosa* | 0.199 | 152 | 65.7 |
| *Pseudocymopterus montana* | 2.113 | 213 | 18.5 |
| *Rosa woodsii* | 4.861 | 182 | 36.2 |
| *Schizachyrium scoparium* | 1.438 | 228 | 75.7 |
| Species | Seed mass | Flowering date | Specific root length |
| *Solidago spp.* | 0.232 | 228 | 63.7 |
| *Sporobolus interruptus* | 1.354 | 228 | 78.9 |
| *Symphyotrichum ascendens* | 0.635 | 243 | 52.7 |
| *Symphyotrichum falcatum* | 0.199 | 259 | 79.1 |
| *Taraxacum officianale* | 0.272 | 228 | 41.2 |
| *Thalictrum fendleri* | 3.088 | 167 | 26.4 |
| *Thlaspi montanum* | 0.564 | 138 | 45.2 |
| *Tragopogon dubius* | 5.078 | 167 | 48.6 |
| *Trifolium longipes* | 1.934 | 197 | 31.5 |
| *Verbascum thapsus* | 0.082 | 182 | 36.7 |
| *Vicia spp.* | 14.516 | 126 | 27.8 |
