# Supplementary material for: An experimental test of the Community Assembly by Trait Selection (CATS) model
Source: PLoS One. 2018 Nov 30;13(11):e0206787. doi: 10.1371/journal.pone.0206787 (PMC6267976; doi:10.1371/journal.pone.0206787)
Supplement: S4 Appendix — (DOCX) [file pone.0206787.s004.docx]

S3 Appendix: Supporting information to the paper

Strahan, R.T. et al. An experimental test of the Community Assembly by Trait Selection (CATS) model

**S3 Appendix**

All traits were measured on robust individuals growing in well-lit environments. Sampling took place in the summer of 2009.

Seed mass is the oven-dry mass of an average seed expressed in mg. When possible, seeds were harvested from several individuals of each species. Seeds housed inside fleshy fruits were removed from the fruiting structures, and if a pappus was present it was removed. Mean seed mass was determined by weighing the total mass of between 20 and 100 individual seeds (depending on the species), then dividing the total dry weight by the number of seeds in the sample (Laughlin et al. 2010).

Specific root length is the ratio of fine root length to dry mass expressed as mg ^-1^. Specific root length was measured on three individuals of each species during the latter part of the growing season. We excavated entire root systems from the soil with shovels and trowels,

and gently washed soil from the roots in the laboratory with clean water rinses. Following the standardized protocol of Cornelissen et al. (2003), the fine (< 2 mm diameter) absorptive roots (i.e. unsuberized, often with evidence of root tips or hairs) were used in our determination of

SRL. The vast majority of the fine roots that we harvested were < 0.5 mm. Root length was measured using the software WINRHIZO V. 2003a (Regent Instruments, Nepean, Ontario, Canada). Roots were then oven-dried for 72 h at 55 _C prior to obtaining dry weights (Laughlin et al. 2010.

Mean Julian flowering dates for each species were determined using regional floras that describe the first and last months that a species is in flower. For example, Artemisia carruthii flowers from August through October, which corresponds to the Julian days 213 through 304. Therefore, the mean Julian flowering day = (213 + 304) ⁄ 2 = 259. Data primarily came from McDougall (1973), but for some species we used data from the Intermountain Flora (Cronquist et al. 1986) or the Flora of North America (Flora of North America Editorial Committee 1993) (Laughlin et al. 2010).

# References

Laughlin DC, Leppert JJ, Moore MM, Sieg CH. A multi-trait test of the leaf-height-seed plant strategy scheme with 133 species from a pine forest flora. Funct Ecol. 2010;24(3):493–501.

Cronquist, Arthur, Noel H. Holmgren, Patricia K. Holmgren, James L. Reveal, and Rupert C. Barneby. 1972. *Intermountain flora; vascular plants of the Intermountain West, U.S.A*. New York: Published for the New York Botanical Garden by Hafner Pub. Co.

McDougall, W. B. 1973.  *Seed plants of Northern Arizona; with keys and detailed descriptions for the identification of families, genera, and species.*  Flagstaff:  Museum of Northern Arizona

Flora of North America Editorial Committee, eds.  1993+.  Flora of North America North of Mexico.  20+ vols.  New York and Oxford.
