# Supplementary material for: An experimental test of the Community Assembly by Trait Selection (CATS) model
Source: PLoS One. 2018 Nov 30;13(11):e0206787. doi: 10.1371/journal.pone.0206787 (PMC6267976; doi:10.1371/journal.pone.0206787)
Supplement: S5 Appendix — (DOCX) [file pone.0206787.s005.docx]

S4 Appendix: Supporting information to the paper

Strahan, R.T. et al. An experimental test of the Community Assembly by Trait Selection (CATS) model

**S4 Appendix**. Generalized Additive Model (GAM) predictions of three

community – weighted mean traits; seed mass , mean Julian flowering date, and

specific root length (SRL). These trait values were used as constraints in the CATS model.

| Environmental treatments | Predicted – Seed mass | Predicted –  SRL | Predicted –  Flowering date |
| --- | --- | --- | --- |
| Shade-basalt | 1.44 | 37.1 | 164 |
| Sun-basalt | 0.82 | 63.5 | 210 |
| Shade-limestone | 1.05 | 52.7 | 178 |
| Sun-limestone | 0.44 | 79.2 | 224 |
