# Supplementary material for: An experimental test of the Community Assembly by Trait Selection (CATS) model
Source: PLoS One. 2018 Nov 30;13(11):e0206787. doi: 10.1371/journal.pone.0206787 (PMC6267976; doi:10.1371/journal.pone.0206787)
Supplement: S6 Appendix — (DOCX) [file pone.0206787.s006.docx]

S5 Appendix: Supporting information to the paper

Strahan, R.T. et al. An experimental test of the Community Assembly by Trait Selection (CATS) model

**S5 Appendix.** Survival (upper diagonal) and germination percentage (lower diagonal) for the five grass species used in the greenhouse experiment. Survival was measured as the proportion of individuals that survived 80 days. Germination rate was measured as the proportion of seeds that germinated at 80 days.

| ^Survival^  _Germination_ | Shade-limestone | Sun-limestone | Shade-basalt | Sun-basalt |
| --- | --- | --- | --- | --- |
| *B. gracilis* | 1  0.34 | 1  0.37 | 0.99  0.38 | 1  0.33 |
| *E. elymoides* | 1  0.18 | 0.86  0.14 | 1  0.20 | 1  0.12 |
| *F. arizonica* | 0.98  0.51 | 1  0.27 | 1  0.53 | 1  0.29 |
| *M. montana* | 0.91  0.14 | 1  0.06 | 1  0.15 | 1  0.12 |
| *P. fendleriana* | 1  0.75 | 1  0.40 | 1  0.80 | 1  0.36 |
